# A new questionnaire for measuring quality of life - the Stark QoL

**DOI:** 10.1186/s12955-015-0367-5

**Published:** 2015-10-26

**Authors:** Jochen Hardt

**Affiliations:** Medical Psychology and Medical Sociology, Clinic for Psychosomatic Medicine and Psychotherapy, Universitätsmedizin Mainz, Johannes Gutenberg Universität, Duesbergweg 6, 55128 Mainz, Germany

**Keywords:** Quality of life questionnaire, Reliability, Construct validity, International research

## Abstract

**Objective:**

The Stark questionnaire measures health-related quality of life (QoL) using pictures almost exclusively. It is supplemented by a minimum of words. It comprises a mental and a physical health component.

**Methods:**

A German sample of n = 500 subjects, age and gender stratified, filled out the Stark Qol questionnaire along with various other questionnaires via internet.

**Results:**

The physical component shows good reliability (Cronbach's alpha = McDonalds Omega = greatest lower bound = .93), the mental component can be improved (Cronbach's alpha = .63, McDonalds Omega = .72, greatest lower bound = .77). Confirmatory factor analysis shows a good fit (Bentlers CFI = .97). Construct validity was proven.

**Conclusion:**

The Stark QoL is a promising new development in measuring QoL, it is a short and easy to apply questionnaire. Additionally, it is particularly promising for international research.

**Electronic supplementary material:**

The online version of this article (doi:10.1186/s12955-015-0367-5) contains supplementary material, which is available to authorized users.

## Introduction

Measurement of Quality of Life (QoL^1^) has become increasingly important in medicine over the past three decades. A search in Pubmed revealed an exponential growth of published articles containing the term "Quality of Life OR QoL" since the 1990s, exceeding 20.000 articles per year since 2013. Randomised controlled trials as well as observational studies increasingly include QoL measures, usually as a secondary endpoint e.g. [1–3]. Additionally, there are studies utilizing measures of QoL as predictors, for example for death [4]. Inclusion of QoL measures into studies is no longer restricted to highly developed western countries, but now includes countries from all over the world e.g. [5, 6].

The Stark QoL is comprised of a total of 16 pictures representing different mood states, energy, social contact, and various physical activities. As far as possible, the content of the items was transferred into the pictures, leaving only very short text elements in between. Fully avoiding text proved to be impossible. A respondent needs to know whether a certain picture displays something (s)he is able to do or something (s)he would like to be able to do or would like to do. Respondents either have to tick the picture in a series that best describes them, or choose a symbol ("--", "-", "0", " + ", "++") near the picture describing how well they are able to perform a certain task. The idea of the questionnaire is based on the Dartmouth COOP Charts [7, 8], where some items similar to those in the Stark QoL were utilized. The name Dartmouth COOP Charts is an abbreviation for a questionnaire used in the Dartmouth-Northern New England Primary Care Cooperative Information Project, and it comprises various dimensions which we would label as aspects of QoL today [9].

When developing the questionnaire, it was important that it could be easily translated into other languages. Since about 10 % of the world’s population are still illiterate [10], it was an additional requirement that respondents who had never learned to read would able to fill it out – naturally after receiving verbal instructions. The Stark QoL is short: it fits on two standard pages. Basically, two components of QoL can be analysed, a mental and a physical one. The questionnaire is called Stark QoL because the pictures were drawn by a German artist named H.P. Stark (www.hans-peter-stark.de/). A first study on 445 students, which focused on the items individually revealed good reliability of the instrument [9].

The aim of the present paper is to present the two dimensions of the questionnaire with various indicators of validity. Therefore, the subscales of the Short Form 36 [11] and the symptom check list 27 plus [12] were compared to the mental and physical component of the Stark QoL.

## Methods

### Sample

A sample of 500 individuals stratified by age and gender filled out the Stark QoL, along with several other questionnaires via internet (http://www.linequest.de). Registered individuals received an email asking them to fill out a questionnaire set containing about 280 items. Participants received compensation of about € 4.30 for filling out the questionnaire. The window was automatically closed after the 500th subject filled out the questionnaire. The ethics commission of the State Chamber of Physicians, Rhineland-Palatinate (Landesärztekammer Rheinland-Pfalz) approved the project (837.185.07). Table 1 displays the sample characteristics. The sample has been described in detail by Hardt et al. [13].

**Table 1:** Sample description (*n* = 500)

Discrete Variables	Categorie	%				
Gender	Female	50.0				
Partnership^a^	Yes	84.0				
School	<9 yrs	13.6				
	10-13 yrs	32.6				
	>13 yrs	53.8				
Occupation	High grade prof.^b^	4.2				
	Lower grade prof.	30.2				
	Skilled non-manual	33.0				
	Skilled manual	5.2				
	Partly skilled worker	10.8				
	Unskilled labourer	6.2				
	Housewife/-husband	10.4				
Continous variables		Range	$\overline{x}$	sd	skewnesss	kurtosis
Item1	Mood	0 - 100	72.60	22.81	−0.74	3.44
Item2	Energy	0 - 100	68.40	46.54	−0.79	1.63
Item3	Social Contact	0 - 100	72.40	30.99	−0.66	2.47
Mental QoL	Score	0 - 100	71.13	26.40	−0.68	2.27
Item4	Shopping	0 - 100	84.35	25.40	−1.64	4.96
Item5	Tying shoe	0 - 100	85.65	23.32	−1.68	5.33
Item6	Taking a glass	0 - 100	90.30	19.98	−2.47	9.34
Item7	Sweeping rubbish	0 - 100	80.85	27.54	−1.37	3.98
Item8	Moving a table	0 - 100	82.75	26.46	−1.58	4.64
Item9	Lifting a heavy box	0 - 100	71.05	31.98	−0.89	2.66
Physical QoL	Score	0 - 100	82.49	25.40	−1.71	5.70
Age	-	18-81	44.82	16.11	0.12	1.80

Note: ^a^partnership lasting 6 months or longer, ^b^abbreviation for "high grade professional"

The sample size was determinated by the plan to perform a confirmatory factor analysis. Muthen and Muthen [14] performed a simulation study using two scales with five items each having factor loadings of .80. Having non-normal data, they recommend a sample size of at least *n* = 265. Hence, a sample size of *n* = 500 should suffice even if some factor loadings were lower here, and the mental component has only three items. For all other statistics the given sample size is fully sufficient. A recent review over 114 patient reported outcome measures found a median sample size of *n* = 207 [15].

### Measures

**The Stark QoL:** The first item measures mood and consists of five smileys, at one end is a very happy face, at the other end a very sad one. Probands were asked to check the one that best applies to them. The second item measures energy and presents two pictures of a person walking, on the left-hand side the walker is full of energy and on the right he seems to be walking almost as if depressed. The third item measures social contact and displays three pictures showing a group of five persons each, one white and four of them grey. The white person symbolizes the proband himself, the grey ones a possible peer group. On one end, the white person is standing in the middle of the group, on the other end alone. Together, these three items constitute the mental component. All items are displayed on one page and are to be answered by making a cross under the picture that best applies to one’s own situation.

On the second page, six items measuring physical functioning are presented. The pictures show activities like carrying a shopping basket, moving a table, tying shoes, etc. Next to each picture, a five point Likert scale was displayed. The text reads "I can", and "++" stands for "very well", " + " for "well", "0" for "fairly", "-" for "poorly" and "- -" for very poorly. Probands are asked to indicate how easily they can perform the activity displayed in each picture. These items constitute the physical component. The whole questionnaire is displayed in Additional file 1.

**The Short Form 36** is a self-rating questionnaire consisting of eight subscales: "Physical Functioning", "Role limitations due to Physical problems", "Bodily Pain", "General Health Perceptions", "Vitality", "Social Functioning", "Role limitations due to Emotional problems", and "Mental Health" [11, 16]. The SF-36 has been translated into more than 40 languages [17].

**The symptom checklist 27 plus** is a six-scale questionnaire. It measures depressive symptoms (current and lifetime), symptoms of social anxiety and agoraphobia, vegetative symptoms and symptoms of pain. The scales (except lifetime depression) assess a time frame of two weeks. They comprise between four and six items each, and have good internal consistencies in population as well as patient samples e.g. [12, 18, 19].

### Statistics

#### Reliability

All items of the present analysis except age and gender were coded between 0 and 100. Scales were calculated as the mean of the items, high values stand for good QoL. The SF-36 was coded according to the manual. The scl-27-plus scales were coded inversely, i.e. high values stand for many and/or severe symptoms. There were no missing data in the survey because the program prompted the respondents to tick any item if one was left blank before changing to the next screen. Since Cronbach’s α [20] as a single measure for reliability is no longer regarded as optimal even by Cronbach himself [21–23], Cronbachs α, McDonalds ω_t_ [24] and the greatest lower bound (glb) [25] were used to estimate reliability.

#### Confirmatory factor analysis

Bentler’s Comparative Fit Index (CFI) [26], the Goodness of Fit Index (GFI), the root mean square error of aproximation (RMSEA), the adjusted Goodness of Fit Index (AGFI), the root mean squared residual (RMR), and the chi square degree of freedom ratio (*X*^2^/df) were reported to assess the overall fit of the confirmatory factor analysis.

#### Construct validity

Pearson correlations were utilized to assess construct validity. In addition to linear effects as represented by the correlation coefficients, all associations between continuous variables were tested for curvy-linearity by including a quadratic term in a regression model. Results for the nonlinear associations are reported as curves in Additional files 2, 3 and 4 if the quadratic term was *p* < .01 (two-tailed), and as a linear regression line otherwise. Statistics were performed by STATA [27], AMOS [28] and the package "psych" in R [29].

## Results

### Item and score distributions

**Fig. 1:**
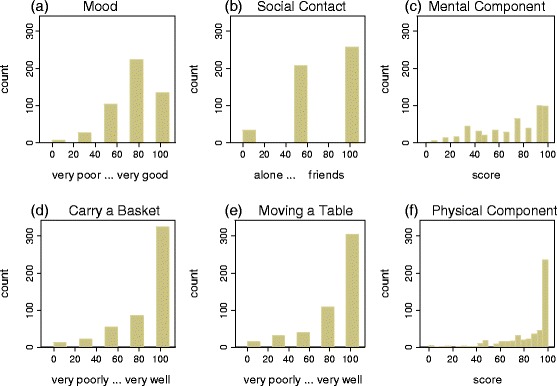
Distributions of items and scores

Figure 1a and b display the distributions of two sample items of the mental component, Fig. 1c the score for mental component. Similarly, Fig. 1d and d the distributions of two sample items of the physical component, Fig. 1f the score. Table 1 shows in the lower half the descriptive statistics all items and scores of the Stark QoL. The mental component has a mean of $\overline{x}$ = 71, the physical component one of $\overline{x}$ = 82. The means of all single items are well above the theoretical midpoint of the scale (50). The easiest item is picking a glass ($\overline{x}$ = 90), the most difficult lifting a box ($\overline{x}$ = 71). All items and scales are left skewed, i.e. subjects are on the positive end of the scale.

### Reliability

Item 9, "lifting a heavy box", did not contribute meaningfully to the physical component. Hence it was not utilized and the score calculated over items 4 – 8. The reliability of the mental component was α = .63, ω_t_ = .72 and glb = .77. For the physical component, it was α = ω_t_ = glb = .93. The items of the mental component had item-rest correlations .40 ≤ r ≤ .62. The items of the physical component had item-rest correlations .77 ≤ r ≤ .85. All items have lower correlations to the foreign scales than to their own.

### Confirmatory factor analysis

**Fig. 2:**
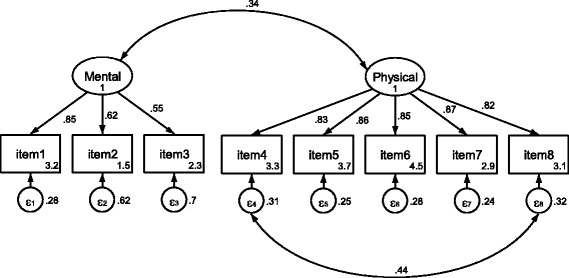
Graphical representation of the Stark QoL with standardizised parameters – i.e. regression coefficients at the single sided arrows, correlation coefficients at the double sided arrows, intercepts in the boxes and residual variances close to the circles

Also in the confirmatory factor analysis, item 9, "lifting a heavy box", did not perform well. The drawing is too similar to item 2, "energy". Hence, it exclusion was confirmed. Additionally, there was a correlation between the errors of item 4, "shopping" and item 8, "moving a table". When the two were allowed to correlate, there was CFI = .97, GFI = .95, RMSEA = 0.094, AGFI = .90, RMR = .034 and χ^2^/df ratio = 5.40. If the two were not allowed to correlate, there was CFI = .94, GFI = .92, RMSEA = 0.124, AGFI = .85, RMR = .037 and χ^2^/df ratio = 8.64 . Standardized factor loadings varied between .55 and .85 in the mental component and between .82 and .87 in the physical component (Fig. 2).

### Correlation between the mental and the physical component

The linear estimate of the association between the mental and the physical component is *r* = .34, *p* < .001 (see Table 2). However, when tested for non-linearity, it can be seen that the association between the mental and the physical component is zero in the lower range and much stronger than *r* = .34 in the higher range (*p* < .001 for the quadratic term). Additional file 3: Figure S1 displays the estimated curve with its 95 % confidence intervals. The latter is narrow in the upper ranger where many observations are, and opens up wide in the lower range. Light blue bubbles display the observed values, big bubbles stand for more subjects than small ones.

**Table 2:** Correlations (Pearson’s r)

	Stark QoL comonent			
	Mental		Physical	
	r	*p*	r	*p*
Stark QoL				
Physical	^a^.34	<.001		
SF-36				
Physical Functioning	^a^.36	<.001	^a^.71	<.001
Role - Physical	^a^.36	<.001	^a^.54	<.001
Bodily Pain	.40	<.001	.57	<.001
General Health	.52	<.001	.53	<.001
Vitality	^a^.66	<.001	.39	<.001
Social Functioning	.54	<.001	.39	<.001
Role-Emotional	.45	<.001	.41	<.001
Mental Health	.63	<.001	.34	<.001
SCL-27-plus				
Depressive Sy, 2 weeks	-.47	<.001	-.18	<.001
Depressive Sy, lifetime	-.31	<.001	-.08	<.060
Vegetative Symptoms	-.28	<.001	-.22	<.001
Agoraphobic Sy	^a^-.32	<.001	-.20	<.001
Sociophobic Sy	-.39	<.001	-.08	<.069
Pain	-.30	<.001	-.29	<.001
Age	^a^-.02	<.726	^a^-.33	<.001
Gender	.08	<.075	.10	<.031

Note: Sy = Symptoms, ^a^indicates that relations are nonlinear, see Additional files 2, 3 and 4

### Validity – SF-36

Table 2 displays the correlations of the two components with the eight scales of the SF-36. Correlations are consistently positive. The **mental component** of the Stark Qol has two high correlations, one to "Vitality" (*r* = .66) and one to "Mental Health" (*r* = .63), three medium correlations to "Social Functioning" (*r* = .54), to "General Health Perceptions" (*r* = .52) and to "Role-Emotional" (*r* = .45). All other correlations are *r* ≤ .40. The **physical component** has one strong correlation to "Physical Functioning" (*r* = .71), followed by three medium ones to "Bodily Pain" (*r* = .57), to "Role-Physical" (*r* = .54) and to "General Health Perceptions" (*r* = .52). All other correlations are *r* ≤ .41. Again, some associations were non-linear as displayed in Additional files 2 and 3.

### Validity – SCL-27-plus

Correlations between the Stark components and the SCL-27-plus are consistently negative and smaller in magnitude. There is one single medium correlation between "current depressive symptoms" and the **mental component** (*r* = −.47), all others are smaller than *r* ≤ .40. The significant non-linear association between "agoraphobic symptoms" and the mental component as well as all linear associations are displayed in Additional files 2 and 4.

### Demographics

The correlation of the **mental component** is close to zero for age, but there is a strong non-linear effect explaining about 1.7 % of the variance of the mental component (see Additional file 4: Figure S2). Young and old participants reported good QoL, middle-aged a relatively poor one (*p* < .003 for the quadratic effect). The **physical component** has a strong negative correlation with age (*r* = −.33). The effect is linear, the added contribution of a quadratic term would contribute non-significantly (p < .812, see Additional file 4: Figure S2). Gender effects are relatively small with point biserial correlations of *r* = .08 for the mental and *r* = .10 for the physical component being non-significant for both components.

## Discussion

### Item and score distributions

In this mainly healthy sample, the answers of all items of the Stark QoL are rather on the side of a high quality of life, leading to left skewed distributions of the scores. This should not necessarily be regarded as negative, the values of the SF-36 show a similar pattern. In research on health related issues, QoL questionnaires are usually designed in theis way, to be able to capture the QoL of severely impaired patients.

### Reliability

The Stark QoL demonstrates a good reliability for its physical component, but the estimates vary strongly for its mental component. The value for α was poor, for ω_t_ acceptable and for glb good. The reason for the poor α lies probably in the fact, that the mental component has three items only. Adding more items would probably improve the scale.

The overall fit of a confirmatory factor analysis was good after freeing one covariance in the physical component, and it still acceptable for the restricted model. Even if Hu and Bentler [30] suggested a cut-off of .95 for the CFI, practice has shown that this criterion cannot always be reached. A second important criterion to evaluate a test was clearly satisfied here: most factor loadings were high. The reason why the two items "shopping" and "moving a table" have a correlated error is unclear and should become examined in further research.

### Validity-SF-36

Construct validity of the Stark QoL was good. The mental component showed the largest correlations to the SF-36 subscales "Vitality" and "Mental Health", two aspects which are explicitly displayed in the Stark QoL. The third item of the mental component assesses social contact – the score had only a moderate correlation to the SF-36 scale "Social Functioning". The physical component of the Stark QoL clearly has the highest correlation to the SF-36 scale "Physical Functioning". Hence, the physical component displays a high specificity. Such an effect would have been expected, because the pictures displaying physical activity partially capture precisely what is asked using words in the items of the SF-36.

### Validity-SCL-27-plus

Construct validity regarding the SCl-27-plus is also good. All correlations were negative, much smaller than those with the SF-36 on average, and some close to zero in the physical component. Such a pattern was expected, because the SCL-27-plus does not assess QoL, but psychological complaints. When the correlations between the mental component and current depressive symptoms versus lifetime depressive symptoms were compared, the one to current depressive symptoms is significantly higher. Hence, one can draw the conclusion that the Stark QoL measures a state rather than a trait.

### Demographics

The effect of age on the physical component is strong, but linear. The expected value for an eighty-year-old is almost 30 points lower than the one for a 20-year-old (exactly 28.59 points). The fact that our physical fitness decreases with age is well-known. However, that the effect was linear here was surprising – usually the decline becomes larger in old age. It can be speculated that this is a consequence of the internet sample which constituted the basis here - possibly only elderly people in good health voluntarily participate in such an internet survey. The estimated value of the mental component is also dependent on age, but here the minimum is at an age of about 50, where the estimated value of mental component is at 68. In comparison, both a 20-year-old and a 75-year-old would receive an estimated value of 80. Gender effects were small and non-significant at p > .01. However, in a further study with the Stark QoL, one should consider these differences.

The present study has the following **limitations:** (1) Data rely on a sample examined via internet. It is not representative for the German population. It is known that about 88 % of Germans and 68 % of Poles have access to the internet [31] – a number which shows that the bias due to sampling procedure should not be too large. On the other hand, the distribution of the variable "years of formal education" displays a pattern which differs clearly from the normal population. (2) This paper reports the construct validity of the questionnaire examined in a relatively healthy sample. It is necessary to conduct studies with patient groups in the future. (3) The Stark QoL was designed to conduct research over various countries including in developing one. Further research on more diverse samples is needed.

## Conclusion

The Stark QoL constitutes an alternative to questionnaires assessing quality of life via worded items. The partly low reliability of the mental component is clearly critical. In a previous study, it received a somewhat better reliability [9]. There are short QoL measures in use e.g. [32, 33]. Even the widely used SF-36 has scales with two and three items [11]. Some authors were satisfied utilizing such short scales, others were more critical. We rather belong to the latter. One or two more items should be included into the mental component of the Stark QoL, we currently think about adding a visual analogue scale for assessing pain and a sort of thermometer with a large heart the top and a small one at the bottom for assessing happiness.

On the other hand, the Stark QoL is a short and efficient measure for two widely assessed dimensions of quality of life and the pictures may make a questionnaire set a bit livelier than one relying solely on worded items. We placed it at the end of the questionnaire. Additionally, translation into many languages should be easy, and international comparisons could be facilitated with the Stark QoL.

## Additional files

Additional file 1:
**The Stark QoL version 1 with nine items and answering options.** (DOC 1221 kb) Additional file 2: Table S1. Tests for nonlinear associations, (unstandardizised regression coefficients). (DOC 54 kb) Additional file 3: Figure S1. Results for nonlinear associations, Stark QoL and SF-36. (DOC 120 kb) Additional file 4: Figure S2. Results for nonlinear associations, Stark QoL and -27-plus. (DOC 94 kb)

## Abbreviations

α: Cronbachs α
AGFI: Adjusted goodness of fit index
CFI: Comparative fit index
GFI: Goodness of fit index
Glb: Greatest lower bound
HRQoL: Health related quality of life
RMR: Root mean squared residual
RMSEA: Root mean square error of aproximation
QoL: Quality of life
Sd: Standard deviation
ω_t_: McDonalds omega
*X*^2^/df: Chi square degree of freedom ratio

## Footnotes

1 There are various forms of QoL. Since this article focuses only on health related QoL, the term QoL stands for health related QoL throughout.
