# Supplementary material for: A new questionnaire for measuring quality of life - the Stark QoL
Source: Health Qual Life Outcomes. 2015 Oct 26;13:174. doi: 10.1186/s12955-015-0367-5 (PMC4621869; doi:10.1186/s12955-015-0367-5)
Supplement: Additional file 1: — The Stark QoL version 1 with nine items and answering options. (DOC 1221 kb) [file 12955_2015_367_MOESM1_ESM.doc]

Additional file 1: The Stark QoL

On this page, you'll find some rows with pictures. Please tick in in each row the one that best describes you in general.

#
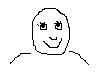

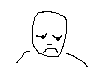

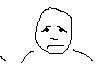

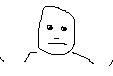

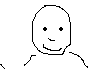
Mood

| **X** | **X** | **X** | **X** | **X** |
| --- | --- | --- | --- | --- |


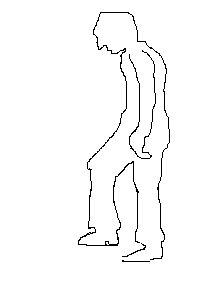

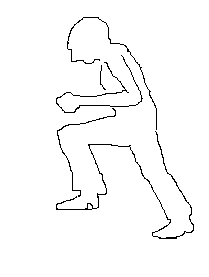
Energy

|  | **x** |  | **x** |  |
| --- | --- | --- | --- | --- |

#
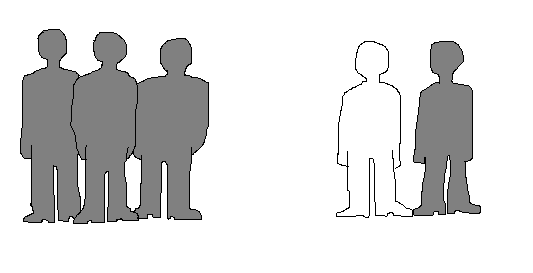

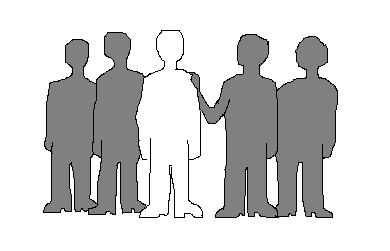

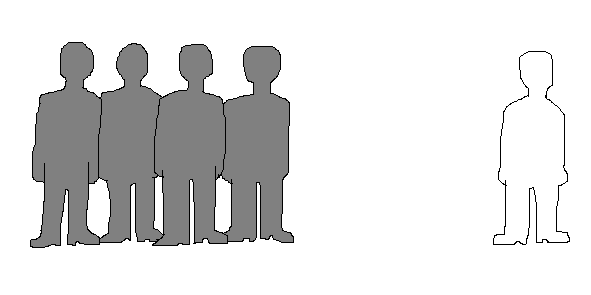
Contact to others

| **X** | **X** | **X** |
| --- | --- | --- |


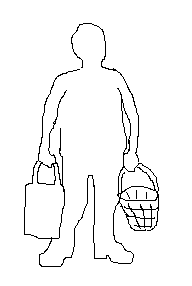
On this page, you'll find pictures displaying several activities. Please tick for each picture if you can do this activity very well (+ +), well (+), fairly (o), poorly (-) oder very poorly (- -).

**
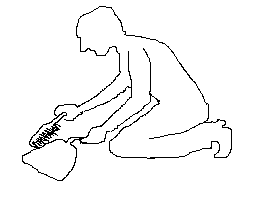

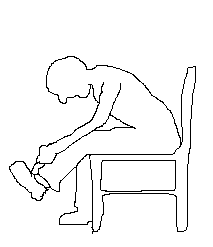

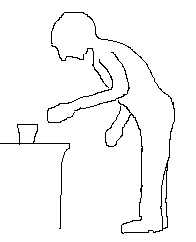
**

**+ +**

**+**

**o**

**-**

**- -**

**+ +**

**+**

**o**

**-**

**- -**

**+ +**

**+**

**o**

**-**

**- -**

**+ +**

**+**

**o**

**-**

**- -**

**
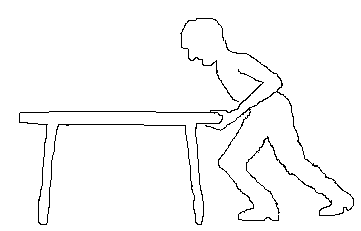

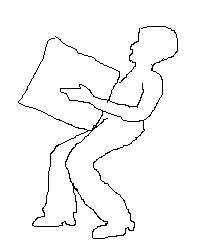
**

**+ +**

**+**

**o**

**-**

**- -**

**+ +**

**+**

**o**

**-**

**- -**


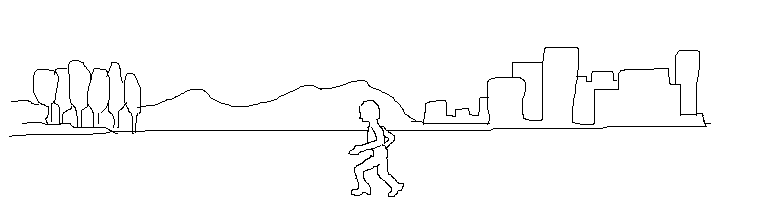
**Thanks!**
