# Supplementary material for: A new questionnaire for measuring quality of life - the Stark QoL
Source: Health Qual Life Outcomes. 2015 Oct 26;13:174. doi: 10.1186/s12955-015-0367-5 (PMC4621869; doi:10.1186/s12955-015-0367-5)
Supplement: Additional file 2: Table S1. — Tests for nonlinear associations, (unstandardizised regression coefficients). (DOC 54 kb) [file 12955_2015_367_MOESM2_ESM.doc]

**Additional file** 2: Tests for nonlinear associations, (unstandardizised regression coefficients)

|  | Stark QoL Comonent (response) | | | | | |  |
| --- | --- | --- | --- | --- | --- | --- | --- |
| Explanatory variable | Mental  Inter. β β^2^ | | | Physical  Inter. β β^2^ | | |  |
| Stark QoL |  |  |  |  |  |  |  |
| Physical | 59.39 | -0.445 | 0.006 |  |  |  |  |
|  |  |  |  |  |  |  |  |
| SF-36 |  |  |  |  |  |  |  |
| Physical Functioning | 51.91 | -0.376 | 0.007 | 42.50 | -0.161 | -0.007 |  |
| Role - Physical | 54.52 | -0.224 | 0.005 | 55.76 | 0.714 | -0.004 |  |
| Bodily Pain | 42.04 | 0.405 | - | 52.41 | 0.451 | - |  |
| General Health | 28.86 | 0.662 | - | 51.99 | 0.513 | - |  |
| Vitality | 2.075 | 1.603 | -0.006 | 61.37 | 0.406 | - |  |
| Social Functioning | 14.80 | 0.666 | - | 53.14 | 0.374 | - |  |
| Role-Emotional | 38.75 | 0.391 | - | 61.59 | 0.280 | - |  |
| Mental Health | 7.939 | 0.898 | - | 58.33 | 0.376 | - |  |
|  |  |  |  |  |  |  |  |
| SCL-27-plus |  |  |  |  |  |  |  |
| Depressive Sy, 2 weeks | 82.21 | -0.607 | - | 87.80 | -0.165 | - |  |
| Depressive Sy, lifetime | 77.52 | -0.236 | - | - | - | - |  |
| Vegetative Symptoms | 81.61 | -0.461 | - | 90.99 | -0.273 | - |  |
| Agoraphobic Sy | 77.49 | -1.127 | 0.010 | 87.06 | -0.281 | - |  |
| Sociophobic Sy | 81.86 | -0497 | - | - | - | - |  |
| Pain | 85.93 | -0.422 | - | 95.52 | -0.306 | - |  |
|  |  |  |  |  |  |  |  |
| Age | 100.0 | -1.409 | 0.015 | 103.9 | -0.426 | - |  |
| Gender | - | - | - | - | - | - |  |

Note: Sy = Symptoms, "-" effect non-significant (p < .01), empty cell: no estimate
