# Supplementary material for: A new questionnaire for measuring quality of life - the Stark QoL
Source: Health Qual Life Outcomes. 2015 Oct 26;13:174. doi: 10.1186/s12955-015-0367-5 (PMC4621869; doi:10.1186/s12955-015-0367-5)
Supplement: Additional file 3: Figure S1. — Results for nonlinear associations, Stark QoL and SF-36. (DOC 120 kb) [file 12955_2015_367_MOESM3_ESM.doc]

**Additional file 3: Results for nonlinear associations, Stark QoL and SF-36**

Legend: The red line represents the estimated association, the grey field its 95% confidence interval. Blue bubbles are the single data points, weighted by their frequency.

Figure S1: Linear and non-linear associations between the Stark QoL and the SF-36.
