# Supplementary material for: A new questionnaire for measuring quality of life - the Stark QoL
Source: Health Qual Life Outcomes. 2015 Oct 26;13:174. doi: 10.1186/s12955-015-0367-5 (PMC4621869; doi:10.1186/s12955-015-0367-5)
Supplement: Additional file 4: Figure S2. — Results for nonlinear associations, Stark QoL and -27-plus. (DOC 94 kb) [file 12955_2015_367_MOESM4_ESM.doc]

**Additional file 4: Results for nonlinear associations, Stark QoL and -27-plus**

Figure S2: Linear and non-linear associations among the Stark QoL and the

symptom check list 27 plus and age.
